# Tissue-Associated Bacterial Alterations in Rectal Carcinoma Patients Revealed by 16S rRNA Community Profiling

**DOI:** 10.3389/fcimb.2016.00179

**Published:** 2016-12-09

**Authors:** Andrew M. Thomas, Eliane C. Jesus, Ademar Lopes, Samuel Aguiar, Maria D. Begnami, Rafael M. Rocha, Paola Avelar Carpinetti, Anamaria A. Camargo, Christian Hoffmann, Helano C. Freitas, Israel T. Silva, Diana N. Nunes, João C. Setubal, Emmanuel Dias-Neto

**Affiliations:** ^1^Medical Genomics Laboratory, CIPE/A.C. Camargo Cancer CenterSão Paulo, Brazil; ^2^Departamento de Bioquímica, Instituto de Química, Universidade de São PauloSão Paulo, Brazil; ^3^Curso de Pós-Graduação em Bioinformática, Universidade de São PauloSão Paulo, Brazil; ^4^Department of Pelvic Surgery, A.C. Camargo Cancer CenterSão Paulo, Brazil; ^5^Department of Pathology, A.C. Camargo Cancer CenterSão Paulo, Brazil; ^6^Laboratory of Molecular Gynecology, Department of Gynecology, Medicine College, Federal University of São PauloSão Paulo, Brazil; ^7^Centro de Oncologia Molecular, Hospital Sirio-LibanêsSão Paulo, Brazil; ^8^Departamento de Alimentos e Nutrição Experimental, Faculdade de Ciências Farmacêuticas, Food Research Center (FoRC), Universidade de São PauloSão Paulo, Brazil; ^9^Department of Clinical Oncology, A.C. Camargo Cancer CenterSão Paulo, Brazil; ^10^Laboratory of Computational Biology and Bioinformatics, A.C. Camargo Cancer CenterSão Paulo, Brazil; ^11^Biocomplexity Institute, Virginia TechBlacksburg, VA, USA; ^12^Laboratory of Neurosciences (LIM-27) Alzira Denise Hertzog Silva, Institute of Psychiatry, Faculdade de Medicina, Universidade de São PauloSão Paulo, Brazil

**Keywords:** mucosa-associated microbiota, rectal cancer, 16S rRNA gene sequencing, *Bacteroides fragilis*, Bacterial diversity and community composition

## Abstract

Sporadic and inflammatory forms of colorectal cancer (CRC) account for more than 80% of cases. Recent publications have shown mechanistic evidence for the involvement of gut bacteria in the development of both CRC-forms. Whereas, colon and rectal cancer have been routinely studied together as CRC, increasing evidence show these to be distinct diseases. Also, the common use of fecal samples to study microbial communities may reflect disease state but possibly not the tumor microenvironment. We performed this study to evaluate differences in bacterial communities found in tissue samples of 18 rectal-cancer subjects when compared to 18 non-cancer controls. Samples were collected during exploratory colonoscopy (non-cancer group) or during surgery for tumor excision (rectal-cancer group). High throughput 16S rRNA amplicon sequencing of the V4–V5 region was conducted on the Ion PGM platform, reads were filtered using *Qiime* and clustered using *UPARSE*. We observed significant increases in species richness and diversity in rectal cancer samples, evidenced by the total number of OTUs and the Shannon and Simpson indexes. Enterotyping analysis divided our cohort into two groups, with the majority of rectal cancer samples clustering into one enterotype, characterized by a greater abundance of *Bacteroides* and *Dorea*. At the phylum level, rectal-cancer samples had increased abundance of candidate phylum *OD1* (also known as *Parcubacteria*) whilst non-cancer samples had increased abundance of *Planctomycetes*. At the genera level, rectal-cancer samples had higher abundances of *Bacteroides, Phascolarctobacterium, Parabacteroides, Desulfovibrio*, and *Odoribacter* whereas non-cancer samples had higher abundances of *Pseudomonas, Escherichia, Acinetobacter, Lactobacillus*, and *Bacillus*. Two *Bacteroides fragilis* OTUs were more abundant among rectal-cancer patients seen through 16S rRNA amplicon sequencing, whose presence was confirmed by immunohistochemistry and enrichment verified by digital droplet PCR. Our findings point to increased bacterial richness and diversity in rectal cancer, along with several differences in microbial community composition. Our work is the first to present evidence for a possible role of bacteria such as *B. fragilis* and the phylum *Parcubacteria* in rectal cancer, emphasizing the need to study tissue-associated bacteria and specific regions of the gastrointestinal tract in order to better understand the possible links between the microbiota and rectal cancer.

## Introduction

The gut microbiota is a vast and diverse ensemble of bacteria and other microorganisms that work together to help digestion, produce vitamins, fatty acids, amino acids and other bioactive compounds, and participate in the regulation of our immune, metabolic, and neurological systems (Shapiro et al., 2014; Boulangé et al., 2016). The understanding of our microbiota, together with the determination of its composition when contrasting healthy vs. diseased states allows the identification of microorganism disturbances that are possibly related to disease development and, therefore, offers a new approach for diagnosis as well as preventive and therapeutic interventions.

Specific dietary components, tobacco and alcohol consumption, which have been linked to the development of a number of pathological states (such as obesity, allergy, diabetes, Crohn's disease, irritable colon syndrome, and cancer) are known to drive microbiome alterations and lead to dysbiosis (Turnbaugh et al., 2009; Leclercq et al., 2014; Allais et al., 2016). The direct action of these elements or of the dysbiosis they cause, appears to be instrumental in the pathogenesis of many diseases and, under certain circumstances, it is possible that dysbiosis may, *per se*, have a direct link with disease development (Duboc et al., 2013). In oncology, studies have been conducted in different neoplastic conditions, identifying roles for specific bacteria in carcinogenesis (Kostic et al., 2012; Riley et al., 2013; Rubinstein et al., 2013), immune evasion (Gur et al., 2015), modulation of the tumor microenvironment (Kostic et al., 2013), and interference with anti-cancer immune responses and immune-surveillance that facilitate chemotherapy activity (Zitvogel et al., 2013; Galluzzi et al., 2015; Vétizou et al., 2015). As a consequence, the emerging concept that cancer needs to be studied considering the complex tumor microenvironment, which includes components such as tumor cells, the surrounding microenviroment and the microbiome, may aid in the development and improvement of cancer treatment, including immunotherapy (Pitt et al., 2016).

Tumors of the lower digestive tract, which include colon and rectal cancer, are among the most prevalent neoplasias worldwide, as well as one of the most fatal. Colorectal cancer (CRC) is the third most commonly diagnosed cancer with 1.4 million people diagnosed annually (Torre et al., 2015). The World Health Organization estimates an increase of 77% in the number of newly diagnosed CRC cases and an increase of 80% in deaths from CRC by 2030 (Binefa et al., 2014). Whereas, colon and rectal cancers have been routinely studied together as CRC, evidences indicate these to be distinct nosological entities. Differences in embryological origin, anatomy, treatment, metastatic potential, and outcome between colon cancer and rectal cancer have led to discussions as to whether neoplastic lesions of these two anatomical sites should be considered as different diseases, with further dichotomization of colon cancers into distal and proximal (Tamas et al., 2015).

The mechanisms involved in sporadic CRC predisposition or development are still poorly understood and the long list of cancer risk factors is continuously expanding and includes age, tobacco, and alcohol consumption, lack of physical activity, increased body weight and, most importantly, diet (Moore and Moore, 1995; Bingham, 2000). Of particular importance is the fact that all these risk factors can directly or indirectly modify the microbiota, making the precise definition of their roles a very complex task. Fecal microbiota studies have contributed greatly in our understanding of the general gut microbiota composition and its dysbiosis in different scenarios (Wu et al., 2013; Sabino et al., 2016). However, possibly due to practical issues related to obtaining the required biopsy samples—from patients and controls—there are still very few available studies focused on the analysis of microbial community compositions of more specific regions of the lower digestive tract, such as the proximal and distal colon, and the rectal tissue. Furthermore, few studies contemplate the fact that fecal- and tissue-associated microbiota are significantly different (Durbán et al., 2011; Hong et al., 2011; Mira-Pascual et al., 2015; Flemer et al., 2016). This fact is particularly relevant for CRC as the intimate crosstalk between the host's epithelium layer and the gut microbial community is a key factor for cell proliferation and development, as well as the regulation of inflammation, a major driver of rectal carcinogenesis (Arthur et al., 2014). Such differences lead to a lack of representativeness with respect to the bacterial biofilm of the rectal mucosa (Durbán et al., 2011; Gevers et al., 2014) and may reflect the disease state but possibly not the tumor microenvironment, which is of great importance to study possible microbiota:disease links.

Here we have addressed such shortcomings by studying the tissue-associated microbiota of 36 subjects, 18 with and 18 without rectal adenocarcinoma. The use of 16S rDNA deep sequencing allowed us to compare non-cancer x cancer mucosa, pointing to specific OTUs and bacterial genera potentially associated with rectal adenocarcinoma.

## Materials and methods

### Cohort

A total of 36 subjects were included after approval by AC Camargo Cancer Center's review board (ACCCC - 1614/11, January 30th, 2012). Tissue biopsies were collected from subjects belonging to one of the following groups:

#### Non-cancer subjects

(Non-Cancer, NC, *n* = 18): All subjects had medical indication of exploratory colonoscopy due to complaints, such as bleeding, abdominal pain, constipation, and chronic diarrhea. No subjects had personal or familial history of colorectal cancer or colitis (either ulcerative, Crohn's, radiation or infectious colitis, chronic inflammatory illnesses), previous colonic or small bowel resection, nor previous colon adenomas or familial polyposis syndrome. Only individuals with complete colonoscopies that allowed the full visualization of the entire colon and showed no significant clinical alterations were included.

#### Colonoscopy and biopsy procedures for the NC subjects

All patients received standard instructions for preparation for colonoscopy that included consumption of 500 ml of mannitol for bowel cleansing, luftal, and bisacodyl. Eligible subjects gave written informed consent to provide colorectal biopsies, had their anthropometric measures taken and answered questions about diet, consumption of alcohol, and tobacco. Colonoscopy was performed using a Pentax videoscope model FC38LX. During biopsy procurement, the rectum was inflated with air and care was taken not to use any suction during advancement of the scope to 7–8 cm from the anal verge. Sterile biopsy forceps were not taken out of the channel of the scope until an area that was completely clear of stool was seen with clear pink mucosa. Biopsies were taken with 2.2 mm sterile standard forceps.

#### Patients diagnosed with rectal adenocarcinomas

(Rectal-Cancer, RC, *n* = 18): Tumor specimens, located in the higher (*N* = 15), mid (*N* = 2), and lower rectum (*N* = 1), were obtained from surgeries to remove the tumor mass. All subjects belonging to this group were recruited at AC Camargo Cancer Center's Pelvic Surgery Department, in São Paulo, Brazil. We included patients that were diagnosed with rectal adenocarcinoma (tumors of stage pT1 or pT2 low- or mid-straight, pT1 or pT2 or pT3 high-straight), that had not undergone any neoadjuvant therapy and had their tumors surgically resected at the Pelvic Surgery Department, AC Camargo Cancer Center, with diagnosis confirmed by the Pathology Department of the same institution. After the histopathologic confirmation of rectal adenocarcinoma diagnosis, surplus samples were macrodissected by an experienced pathologist and used for DNA extraction and bacterial community profiling. *Exclusion criteria* were: Patients subjected to neoadjuvant therapy prior to tissue collection; patients reporting inflammatory bowel diseases or with hereditary cancer syndromes. We also excluded all subjects (cases and controls) who reported the use of antibiotics for at least 4 weeks prior to sample-collection.

### DNA extraction

DNA extraction started after incubating the samples for 18 h in 600 μl of a lysis buffer (Qiagen) and 15 μl of proteinase K (20 μg/μl) at 55°C. After this period, DNA samples were extracted using a standard phenol chloroform protocol, followed by ethanol precipitation, quantification using a spectrophotometer (Nanodrop—Thermo Scientific), and visualized on 2% agarose gels to inspect DNA integrity.

### PCR amplification and sequencing of the V4–V5 region of 16S rRNA gene

#### Oligonucleotide primer selection and coverage analysis

The V4–V5 region was amplified using a primer set designed to generate amplicons compatible with the chemistry available for the Ion Torrent PGM platform, that allowed ~400 nt of high quality sequences (Ion PGM Sequencing 400 Kit). Coverage of the primer set was evaluated using the Ribosomal Database Project's (RDP—Release 11.2) ProbeMatch (Cole et al., 2014) and the ARB Silva's (Release 115) TestPrime (Klindworth et al., 2013). The forward primer (5′-AYTGGGYDTAAAGNG-3′) and reverse primer (5′-CCGTCAATTCNTTTRAGTTT-3′) corresponded to positions 562 and 906, respectively, of the *Escherichia coli* 16S rRNA gene.

#### PCR amplification and amplicon sequencing

Three 50 μl amplification replicate reactions were performed per sample, each containing: 2.5 μM of each primer; 25 μl of Kapa Hotstart High Fidelity Master Mix (Kapa Technologies) and 25 ng of genomic DNA (gDNA). Thermocycling conditions were: 95°C, 3 min; 98°C, 15 s, and 40°C, 30 s for 35 cycles; followed by a last extension step at 72°C for 5 min. Amplicons of the three reactions from each subject were pooled and purified using a MinElute PCR Purification Kit (Qiagen). The purified products were run on 1.5% agarose gels and gel bands within the expected amplicon range were excised using sterile and disposable scalpels and purified using the Qiaquick gel extraction kit (Qiagen) to remove artifacts, primer-dimers and non-specific bands. Amplicons were end-repaired and Ion Torrent adaptors with barcodes were ligated. Equimolar amounts of amplicons from each sample were pooled, using the Ion Torrent qPCR quantitation kit (Thermo Scientific, Carlsbad, USA), and used for emulsion PCR. All samples were sequenced on the Ion torrent PGM platform (Thermo Scientific, Carlsbad, USA) using two 318 v2 chips. Samples from both groups were processed simultaneously, to avoid possible batch effects.

### Sequence analysis

#### Sequence filtering

Sequences processed by the latest version of the Ion Torrent server (v3.6.2) were used as input into the *Qiime* (*Quantitative insights into microbial ecology*) software package (Version 1.6.0) (Caporaso et al., 2010a). We first removed sequences with an average quality score <20 using a 50 nt sliding window. Then, we identified barcodes used for subject-assignment, allowing a maximum of 2 mismatches, and discarded sequences with no barcodes, and <200 nt or >500 nt after barcode removal. PCR primers identified at the start or at the end of the reads, allowing a maximum of 4 nt mismatches, were trimmed and sequences with no identifiable primers were discarded. After primer trimming we removed all sequences below 200 nt and the remaining sequences were used as input for downstream analysis.

#### Sequence clustering and OTU filtering

Filtered sequences were clustered with 97% identity using UPARSE (implemented in USEARCH v7) (Edgar, 2013) and the seed sequence of each cluster was picked as a representative. Chimeric sequences (and clusters) were identified using UCHIME (Edgar et al., 2011) and the Broad Institute's chimera slayer database (version microbiomeutil-r20110519) and excluded from further analysis. The RDP classifier (Wang et al., 2007), as implemented within the *Qiime* interface (default parameters), was used to assign taxonomic ranks using a minimum confidence value of 80% and, subsequently, to each operational taxonomic unit (OTU). Unless otherwise stated, OTUs that occurred in less than 25% of all samples and with less than 3 reads were not considered.

### Alpha and beta diversity analysis

We rarefied the OTU table to 17,414 sequences per sample in order to calculate species diversity, using the Shannon-Weaver index (Shannon, 1948) and the Simpson index (Simpson, 1949), and richness (by using the observed species) implemented in the R Phyloseq package (McMurdie and Holmes, 2013).

For beta diversity analysis, OTU-representative sequences were aligned using PyNAST (Caporaso et al., 2010b) against the aligned green genes core set (DeSantis et al., 2006) with *Qiime* default parameters, and the alignments were lanemask-filtered (Lane, 1991). A phylogenetic tree was built using FastTree (Price et al., 2009), weighted and unweighted UniFrac (Lozupone and Knight, 2005) distances were calculated and a distance matrix was generated. Using the R phyloseq package, distance matrices were used to calculate coordinates for principal coordinate analysis (PCoA).

#### Enterotypes

Community types of each sample were analyzed by the Dirichlet multinomial mixture model-based method (Holmes et al., 2012) using rarefied genera level counts of 16S rRNA sequencing reads. Partioning around medoids (PAM) enterotyping was performed in R using genera level relative abundances and the “cluster” package (Maechler et al., 2015). We applied 4 distance metrics: Weighted UniFrac, Unweighted UniFrac, root Jensen-Shannon divergence, and Bray-Curtis and assessed the quality of the clusters using prediction strength (Tibshirani and Walther, 2005), silhouette index (Rousseeuw, 1987), and the Caliński-Harabasz statistic (Calinski and Harabasz, 1974) using the “fpc” R package (Hennig, 2015).

### Differential abundance analysis

To investigate differences in OTU, phyla and genera abundances between both groups, raw counts were normalized then log transformed using the normalization method below, as performed by a previous study (Sanapareddy et al., 2012):

$$\begin{array}{l} Normalized\;count=\text{ ​​}\log_{\text{10}}((\frac{raw\;count}{number\;of\;sequences\;in\;that\;sample})\\ \;\times \text{ ​}average\;number\;of\;sequences\;per\;sample\text{​}+\text{​}1) \end{array}$$

We also evaluated high-level phenotypical differences in microbial composition between both groups. Quality control passed sequences were closed-reference picked at 97% identity using UCLUST_Ref (Edgar, 2010) and the green genes core set (Version 13.5). The resulting OTU table was rarefied to 13,944 sequences and submitted to BugBase (http://github.com/danknights/bugbase) in order to calculate differences between both groups in terms of microbial phenotypes.

### Data validation

#### Digital droplet PCR of bacteroides fragilis 16S rRNA

We detected and quantified the absolute number of 16S rRNA *B. fragilis* copies in our samples using the *QX200*™ *Droplet Digital*™ *PCR System* (Bio-Rad). The primers used to amplify the *B. fragilis* 16S rRNA gene were: BF-fwd 5′-TCRGGAAGAAAGCTTGCT-3′ and BF-rev 5′-CATCCTTTACCGGAATCCT-3′(Tong et al., 2011) and to ensure further specificity, a labeled probe BF-p 5′(FAM)-ACACGTATCCAACCTGCCCTTTACTCG-3′ (BHQ1) (Tong et al., 2011) was included in the reaction. We used a commercial RNAseP *Copy Number Reference Assay* (Thermo-Fisher) to detect and quantify human DNA. Microdroplets (≈20.000/reaction) were generated on the Bio-Rad *QX-100* following the manufacturer's instructions. RNAse P and *B. fragilis* ddPCR were performed in 96 well-plates, in a final volume of 20 μl, containing: 15 ng of total DNA, 10 ul of ddPCR supermix for probes (Bio-Rad), 8 pmol of each PCR BF-primer and 2 pmol of the BF-probe, or 1 μl of RNAse P assay. PCR conditions were: 50°C- 2 min; 95°C- 10 min; 95°C- 15 s and 60°C- 1 min for 40 cycles. After cycling, the 96-well plate was immediately transferred on a *QX200 Droplet Reader* (Bio-Rad), where flow cytometric analysis determined the fraction of PCR-positive droplets vs. the number of PCR-negative droplets in the original sample. Data acquisition and quantification was carried out using *QuantaSoft Software* (Bio-Rad). To ensure the accuracy of the results, a minimum of 10,000 acceptable droplets per reaction were required for quantification using the *QuantaSoft software*. Samples yielding a minimum of 3 positive droplets from 10–15,000 droplets analyzed were scored as positive.

### Immunohistochemistry

Immunohistochemistry was performed in an automated Benchmark platform (Ventana Medical Systems, USA) for Anti-*B. fragilis* LPS antibody (mouse monoclonal—Abcam 1265/30) in whole slide tissues. Alkaline phosphatase conjugated to secondary polymeric system was used for IHC visualization. The selection of positive and negative samples was guided by the high-throughput sequencing (HTS) data and used to confirm the presence of *B. fragilis* in the sample set. The primary antibody was omitted to evaluate background staining.

### Statistical analysis

Wilcoxon tests were used to compare mean differences between tumor and biopsy samples for phyla, genera and OTU log-abundances. Considering t = total number of taxa tested, *p* = raw *p*-value and R = sorted rank of the taxon, *P*-values were corrected for multiple testing (Sanapareddy et al., 2012) using:

$$\begin{array}{l} Adjusted\;p\;value\;=\;\frac{t\;\times \;p}{R} \end{array}$$

Fold changes for each genera/OTU were calculated using:

$$\begin{array}{l} Log\text{2}FC\;=\;\log_{\text{2}}\left(RC\;average\;+\;1\right)\;-\;\log_{\text{2}}\left(NC\;average\;+\;1\right) \end{array}$$

Chi-Square tests were performed on subject's categorical data such as gender, alcohol and tobacco use and vital status. Student *t*-tests were performed to compare differences in the means between both groups for age, height, weight, BMI, and alpha diversity. We used ANOSIM and ADONIS (Oksanen et al., 2016) to compare differences in beta-diversity between groups using 3 distance metrics weighted UniFrac, unweighted UniFrac and Bray-Curtis for categorical, and numerical variables, respectively. Linear models were built using normalized counts at the genera and OTU level to investigate associations with clinical-pathological characteristics of rectal-cancer samples, such as lymph node and perineural neoplastic invasion status. Unless otherwise stated, values were reported as mean ± *SD* (standard deviation) and *P*-values <0.05 were considered statistically significant. All calculations were performed within the R statistical computing environment (R Foundation, 2011) unless otherwise stated.

## Results

### Subjects and tissue sample characteristics

We analyzed tissue-associated bacteria from mucosal biopsies of 18 non-cancer controls and 18 rectal adenocarcinoma tumors using 16S rRNA high throughput amplicon sequencing. We found no significant differences between rectal-cancer and non-cancer subjects regarding age and gender distribution, tobacco, and alcohol use and other risk factors (Table 1). All samples consisted of rectal-biopsies. The biopsies of individuals with no tumor lesions derived from the mid rectum and were distributed along the ~12 cm-long human rectum, with most samples deriving from the higher-mid rectum (94%).

**Table 1:** **Subject and sample data**.

**Demographic**	**Non-cancer (*n* = 18)**	**Rectal-cancer (*n* = 18)**	***P*-value**
Age	55.2 ± 15.7	59.3 ± 8.8	0.348
**GENDER (%)**			
Female	9 (50)	8 (44)	1
Male	9 (50)	10 (56)	
Height	1.65 ± 0.08	1.70 ± 0.09	0.1
Weight	73 ± 14.1	73.8 ± 13.5	0.87
BMI	26.6 ± 3.7	25.3 ± 3.6	0.29
**ALCOHOL USE (%)**			
Yes	8 (44)	5 (28)	0.568
No	10 (56)	12 (67)	
Undetermined	0 (0)	1 (5)	
**TOBACCO USE (%)**			
Yes	12 (67)	6 (28)	0.129
No	6 (33)	11 (62)	
Undetermined	0 (0)	1 (5)	
**Pathological tumor size staging (%)**			
pT2	N.A.	5 (28)	N.A.
pT3		13 (72)	
**Pathological lymph node metastasis staging (%)**			
pN0	N.A.	11 (62)	N.A.
pN1		3 (16)	
pN2		4 (22)	
**Distant metastasis staging (%)**			
M0	N.A.	18 (100)	N.A.
**Invasion (%)**			
Perineural	N.A.	4 (22)	N.A.
Angiolymphatic		14 (78)	
**Vital status (%)**			
Alive	18 (100)	17 (95)	1
Deceased	0 (0)	1 (5)	

N.A., Not applicable.

### Primer coverage

Our analyses indicate that the PCR primers used here (V4–V5 region of the 16S rRNA gene) cover 84.4 and 52.1% of all eubacterial sequences present in the ARB SILVA database and the Ribosomal Database Project, respectively (Supplementary Table 1). Coverage rates were evenly distributed among most bacterial phyla, except for *Verrucomicrobia*, where coverage rates were 21 and 10.9%, dropping below the 75 and 48% averages of taxa present in the SILVA and RDP databases, respectively.

### Sequence analysis

#### Sequence generation and filtering

A total of 12,078,140 sequence reads were generated, with a mean sequence length of 304.5 ± 97.34 nt (standard deviation—std). After quality filtering and primer trimming, 5,593,020 (46.3%) sequences remained, with an average of 155,361 sequences/sample and a mean sequence length of 315 ± 30 nt.

#### Sequence clustering and OTU filtering

When all individuals were considered, a total of 3222 OTUs were obtained. Thirty-one (0.7%) OTUs were identified as chimeras by UCHIME and 209 (4.7%) could not be assigned to a taxonomic rank. After filtering OTUs with less than three sequences and not present in at least 25% of all samples (NC and RC combined), 1492 OTUs remained.

### Alpha and beta diversity

#### Species richness and diversity

**Figure 1:**
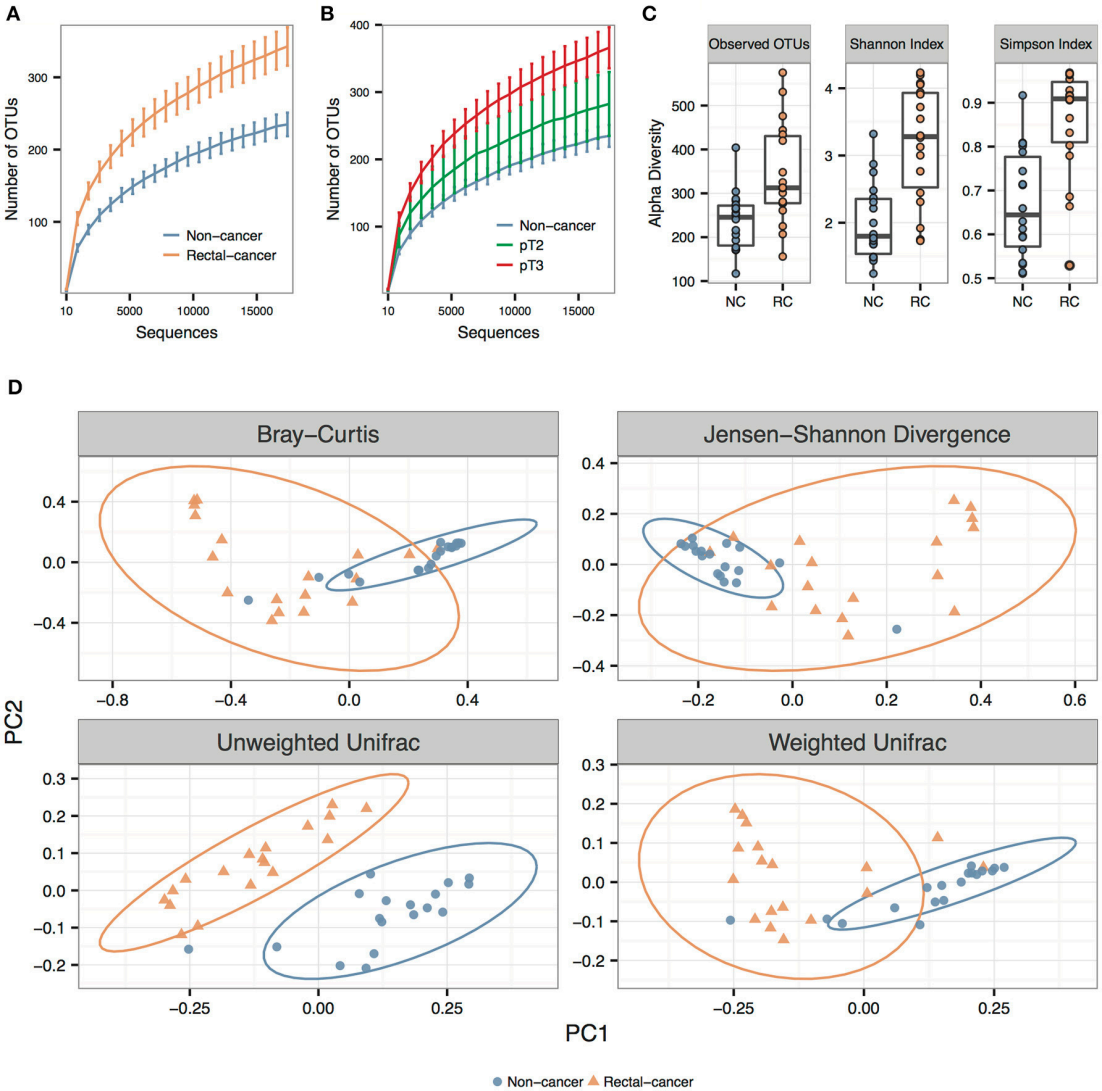
**Alpha and beta diversity for non-cancer and rectal-cancer samples**. **(A)** Rarefaction curves showing the average number of observed OTUs for both groups. Error bars represent ± standard error of the mean. Blue: Non-cancer samples; red: Rectal-cancer samples. **(B)** Rarefaction curves showing the average number of observed OTUs for NC samples and for smaller (pT2) and larger rectal tumors (pT3). Error bars represent ± standard error of the mean. **(C)** Boxplots showing alpha diversity in rectal-cancer samples and non-cancer samples using different metrics (Observed OTUs, Shannon index and Simpson index). **(D)** Principal Coordinate Analysis (PCoA) ordination plots for four distance metrics (Bray-Curtis, Jensen-Shannon Divergence, Weighted and Unweighted UniFrac). Ellipses represent the 95% confidence level assuming a multivariate t-distribution.

We observed significantly higher species richness and species diversity in rectal cancer samples compared to controls. This was observed for the number of OTUs, the Shannon index and the Simpson Index (*P*-values = 0.002, <0.001, and <0.001, respectively) (Figures 1A,B). When we stratified rectal-cancer samples into smaller (pT2) and larger tumors (pT3), we observed an increase in species richness, with an average of 280 and 366 OTUs, respectively, compared to 236 OTUs in NC; however this effect reached no statistical significance between pT2 and pT3, maybe because of the reduced number of pT2 samples (*N* = 5, compared to *N* = 13 for pT3) (Figure 1B and data not shown).

#### Beta diversity

Using three distance metrics we observed consistent and statistically significant differences between the sample groups when considering cancer status (Bray-Curtis, Unweighted and Weighted UniFrac; *p*-value: 0.001; ANOSIM using 999 permutations), but not for any other categorical or numerical variable, which included amplicon library construction, age, gender, BMI, alcohol, and tobacco use (Figure 1D; Supplementary Table 2).

#### Enterotypes

**Figure 2:**
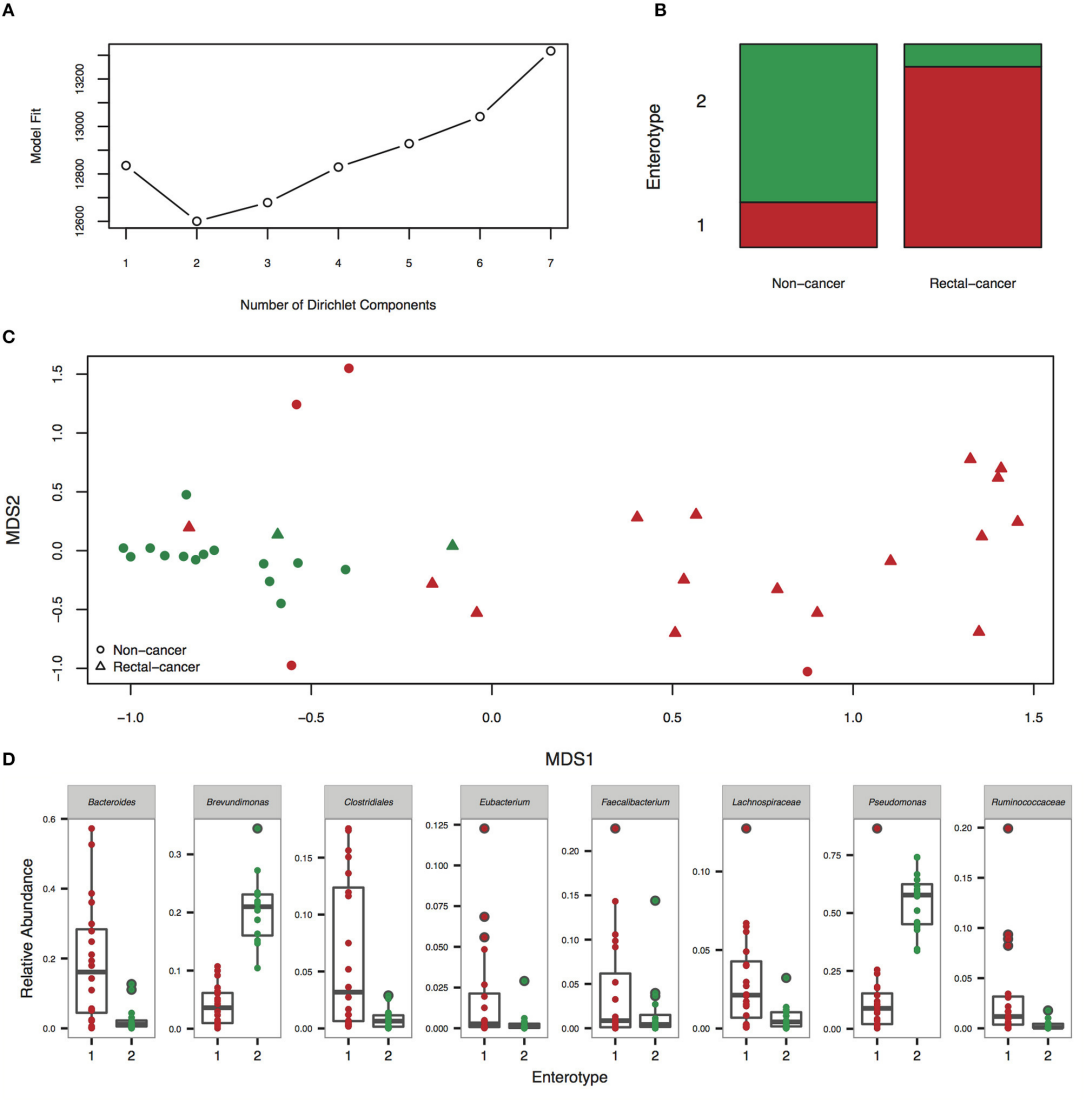
**Enterotyping analysis reveals the presence of two community types**. **(A)** Fitting to the Dirichlet Multinomial Mixture model indicates optimal classification into two community types. **(B)** Distribution of rectal-cancer samples and non-cancer samples in both enterotypes (*P* = 0.0001, Fisher's exact test). **(C)** Non-metric dimensional scaling (NMDS) ordination plot of Jensen–Shannon divergence values between samples. Red, community type-1; green, community type-2. **(D)** Relative abundances of the top 8 most abundant genera in the two community types.

Enterotyping analysis using a Dirichlet multinomial mixture model divided our cohort in two clusters (Figures 2A–C). Enterotype I was significantly enriched for rectal-cancer samples, whilst enterotype II was composed mostly of non-cancer samples (*p*-value: 0.0001, Fisher's exact test). Enterotype I had higher abundances of *Bacteroides, Clostridiales, Dorea*, and other genera, whilst enterotype II was characterized by elevated amounts of *Pseudomonas* and *Brevundimonas* (Figure 2D). When using the PAM based enterotyping method and criterion adopted by a meta-analysis of human enterotypes (Koren et al., 2013), we found two enterotypes with prediction strength above 0.9 (meaning that 90% of the data points fall within the cluster and 10% are outliers) using the Weighted UniFrac distance (Supplementary Figure 1).

### Global signatures of the microbial community

#### Phyla log abundances

We observed a significant difference in the log abundances of 6 out of 12 detected phyla between both groups (Supplementary Figure 2). The most abundant phyla identified were (in decreasing order) *Proteobacteria, Firmicutes, Bacteroidetes, Fusobacteria, Actinobacteria*, and *Verrucomicrobia*. In non-cancer samples, we observed higher log abundances of *Actinobacteria, Cyanobacteria, Proteobacteria*, and *Planctomycetes*, whose presence was detected in 9/18 NC samples, with an average log abundance of 0.54 and was absent from all RC individuals (*p*-value < 0.001). In rectal-cancer we found greater log abundances of *Bacteroidetes* and of the much less known candidate phylum *OD1* (also known as *Parcubacteria)*, whose presence was detected in 14/18 RC samples with an average log abundance of 0.71 vs. 1/19 NC samples and an average log abundance of 0.02 (*p*-value < 0.001).

#### Genera log abundances

**Figure 3:**
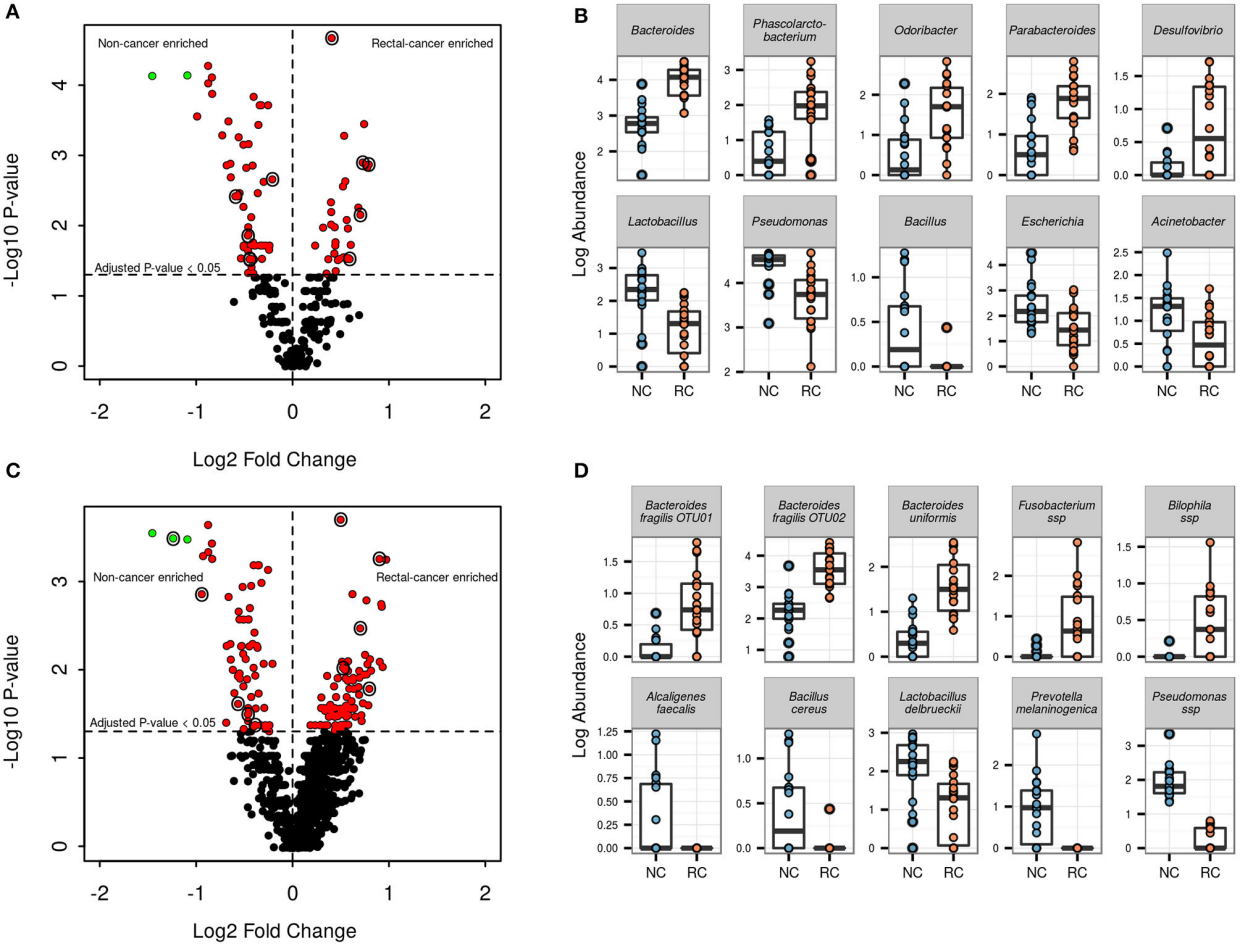
**Genera and OTU level differential abundance signatures**. **(A)** Volcano plot for all 260 genera found in our samples. Red points indicate genera with an adjusted *p*-value <0.05; green points indicate genera with an adjusted *p*-value <0.05 and log_2_FC > 1. Points circled in black are genera shown in the adjacent boxplot. **(B)** Boxplots showing log abundances for 5 genera with significant increases (top) and 5 genera with significant decreases in rectal-cancer samples (bottom). **(C)** Volcano plot for 1492 OTUs found in our samples. Points color scheme is the same as in **(A). (D)** Boxplots showing log abundances for 5 OTUs with significant increases (top) and 5 OTUs with significant decreases in rectal-cancer samples (bottom).

At the genus level, 86 out of 260 genera (33%) showed significant differential log abundances between both groups (Figure 3A and Supplementary Table 3). The top five genera with differential log abundances between the groups were *Bacteroides, Phascolarctobacterium, Odoribacter, Parabacteroides, Desulfobrio* (more abundant in the cancer group), and *Lactobacillus, Pseudomonas, Bacillus, Escherichia, Acinetobacter* (more abundant in the non-cancer set) (Figure 3B).

#### OTU log abundances

Of the 1492 OTUs identified, 163 (10.9%) were found to have significant differential log abundances between both groups (Figure 3C). Three OTUs assigned to the genus *Bacteroides, two* belonging to *B. fragilis* and one to *B. uniformis*, as well as OTUs assigned to *Bilophila* sp. and *Fusobacterium* sp., were significantly more abundant in rectal-cancer samples (Figure 3D). In non-cancer samples, OTUs assigned to *Alcaligenes faecalis, Bacillus cereus, Lactobacillus delbruecki, Prevotella melaninogenica* and *Pseudomonas* ssp had higher log abundances compared to rectal-cancer samples. Four OTUs belonging to the *Bacilli* class were more abundant among non-cancer samples, including *L. delbrueckii* (Figure 3D).

When analyzing high-level phenotypical differences, the most striking differences included a higher abundance of anaerobic bacteria and a deficit in biofilm-forming bacteria in rectal-cancer samples (Supplementary Figure 3). In our searches for associations between rectal-cancer samples' clinical data and genera/OTU log abundances using linear regression, we found significant associations between genera/OTUs with regards to the presence of lymph node disease (Supplementary Table 4) and perineural invasion (data not shown). We found a significant increase of *Coprococcus, Dorea, Roseburia*, and *Mogibacterium* in lymph node positive rectal-cancer (Supplementary Figure 4).

### ddPCR confirms the higher counts of *B. fragilis* in tumor samples

**Figure 4:**
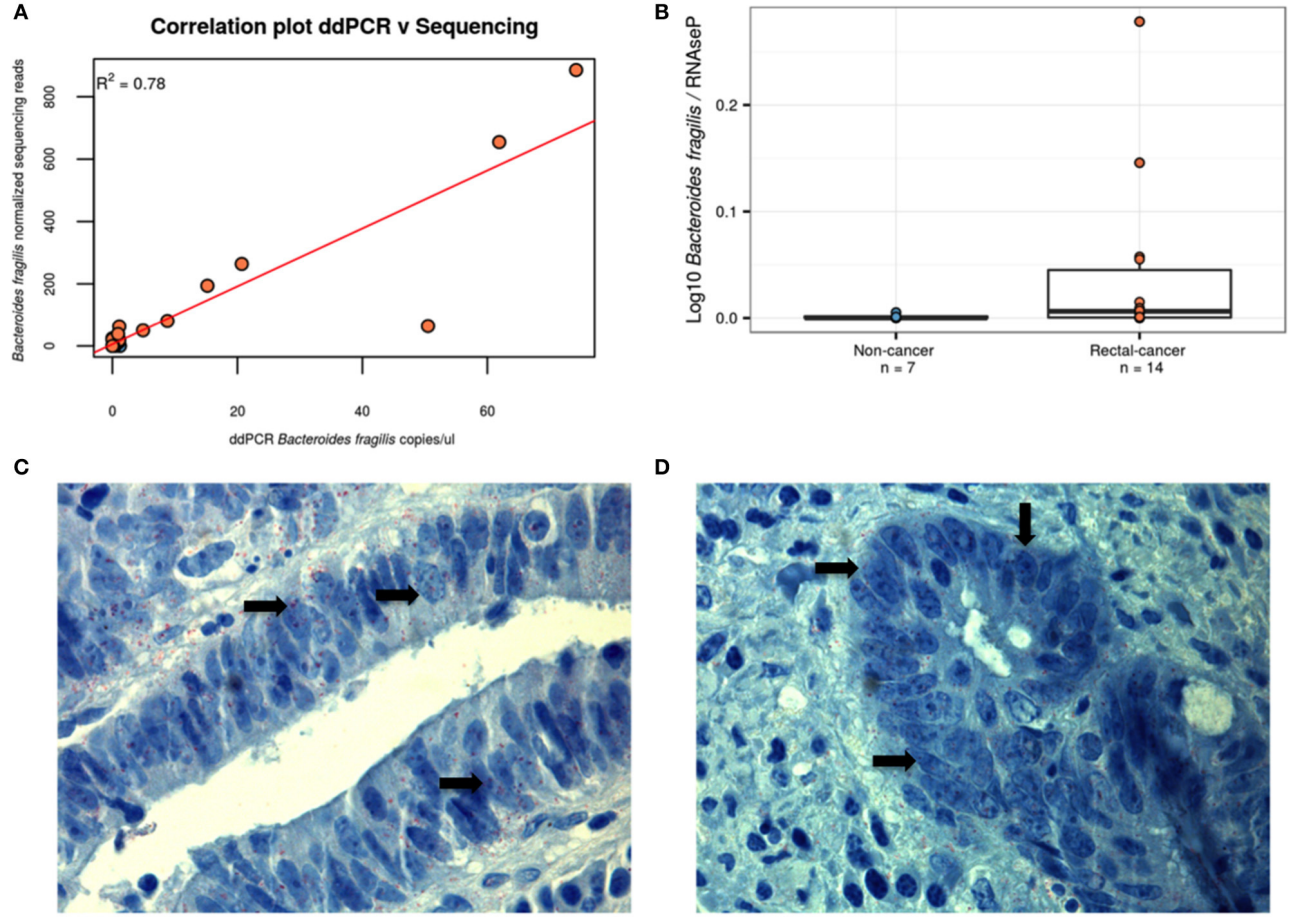
**Alternative approaches demonstrating the presence of *B. fragilis***. **(A)** *B. fragilis* ddPCR quantification correlates with HTS-derived data. Using linear regression we obtained a correlation of *R*^2^ = 0.78, *P* < 0.001. Blue: Non-cancer samples; red: Rectal-cancer samples. **(B)** Boxplot showing log10 of the ddPCR ratio found for *B. fragilis* after normalizing for RNAseP values for both groups. Blue: Non-cancer samples; red: Rectal-cancer samples. **(C,D)** Immunohistochemisty analysis of two *B. fragilis*-positive rectal-cancer samples, demonstrating the presence of this microbe (antibodies are labeled in red and shown with arrows) using magnification of 1000X.

As two OTUs classified as *B. fragilis* were among the smallest *p*-values found and with the highest fold change between the groups, we designed a specific ddPCR assay for *B. fragilis* in order to verify the validity of the results using an alternative approach. As can be seen in Figures 4A,B, we observed the expected correlation (*R*^2^ = 0.78) between both methods and confirmed the higher ratio of *B. fragilis*/human DNA in rectal cancer samples, validating the results of our sequencing approach (*P*-value = 0.04, Wilcoxon Rank-Sum Test). To further evidence the presence of *B. fragilis* in tumor specimens, we performed an immunohistochemistry assay on 3 rectal-cancer samples using an anti-*B. fragilis* LPS antibody and found that this bacterium was present in rectal-cancer tissue (Figures 4C,D).

## Discussion

In face of the microbiota gradient found in the human digestive tract (Zhang et al., 2014; Gao et al., 2015; Flemer et al., 2016) and the possibility that tissue-associated microorganisms could play a more direct role in immunomodulation and cancer development, we investigated bacterial populations present in tissue biopsies, which may be relevant to pathological processes. Instead of studying colon and rectum samples together, our work is more specific as it is focused and contains only rectal tumors. Whereas, we achieved high 16S rRNA coverage from a large spectrum of bacteria from cancer samples, before any therapeutic intervention, we also see limitations, such as our relatively small sample size of 36 individuals. However, effect size analysis (Kelly et al., 2015) between both groups revealed an ω^2^ ranging from 0.13 to 0.26, depending on the metric of pairwise distance, with PERMANOVA *p*-values <0.001 and power of 1 (data not shown), a finding that indicates that this sample size allows the observation of significant microbial differences between our two sample groups. We also need to point out that, whereas the primer pair used here gives a good coverage of most phyla, it has a poor coverage of the two closely related bacteria phyla *Lentisphaerae* and *Verrucomicrobia*.

In our study, we observed increased species-diversity and -richness among rectal-cancer samples. Higher species-diversity and -richness were seen in rectal tissue samples from adenomas compared to normal samples (Sanapareddy et al., 2012) and CRCs vs. adenomas (Nakatsu et al., 2015) and increased richness was found in CRCs compared to both adenomas and controls (Mira-Pascual et al., 2015). However, when looking at fecal samples, studies have had conflicting results. One study found increased diversity of both genes and genera along the adenoma-carcinoma transition (Feng et al., 2015), whereas another found a decrease in diversity when comparing carcinoma samples and normal controls (Ahn et al., 2013) and a third found no differences between controls, adenomas and carcinomas (Zeller et al., 2014). It is noteworthy to state that these fecal studies grouped proximal and distal colon cancers together with rectal cancers, which could have led to differences in their results. We should note that the five cases of early-stage lesions (pT2) showed, on average, intermediate microbial richness, when compared to non-cancer biopsies and a more advanced neoplastic stage (pT3). This suggests that increased species richness of cancer lesions could have an early role in rectal carcinogenesis.

Inter-individual microbial community heterogeneity of the human gut is influenced by spatial distribution, micro-heterogeneity, host genetics, dietary preferences, and mucin content (Eckburg et al., 2005; Hong et al., 2011; Zhang et al., 2014), and has posed a long-standing challenge when investigating microbial signatures implicated in CRC tumorigenesis. However, our results show that despite the high inter-individual differences, a common microbial community pattern appears to emerge, as shown in the PCoA analysis that clustered non-cancer and rectal-cancer groups separately (Figure 1D), suggesting a common dysbiotic setting related to this neoplasia.

We performed a global analysis of high-level phenotypical differences for bacteria identified in both groups. We highlight the higher abundance of anaerobic bacteria in the RC group in agreement with a previous study (Warren et al., 2013) and the reduction of biofilm-forming bacteria. The latter is a finding that may point to barrier breakage that would contribute to rectal colonization by relevant bacteria (Reid et al., 2001) (Supplementary Figure 3).

The alterations we found at the phylum level include higher levels of *Cyanobacteria* (possibly *Melainabacteria*) (Soo et al., 2014), *Actinobacteria, Bacteroidetes, OD1, Proteobacteria*, and *Planctomycetes* in the RC-group. We should note an important abundance difference for bacteria of the candidate phylum *OD1* (*Parcubacteria*). These highly adapted organisms have not been isolated *in vitro* yet; they have small genomes (<1 Mb) and reduced metabolic properties identified in a range of anoxic environments. The absence of biosynthetic capabilities and DNA repair enzymes, derived from the genomic analyses of some *OD1* bacteria, suggests a role as ectosymbionts (Nelson and Stegen, 2015). However, the putative role of these microbes in rectal cancer remains to be determined. A second phylum, *Planctomycetes*, which are atypical bacteria (Fuerst and Sagulenko, 2011) relatively close to *Verrucomicrobia* (Hou et al., 2008) and more frequently observed in aquatic environments (such as saltwater, fresh water, and acidic mud), also showed potential as a biomarker for RC, with striking differences between the groups.

Interestingly, our study also indicated the differential abundance of more specific microbes after comparing NC and RC groups. *B. fragilis*, a symbiotic organism common to the human intestinal tract, was found to be more abundant in rectal-cancer samples seen by 16S rRNA HTS and confirmed by ddPCR. Other studies that investigated tissue-associated bacteria also found increased abundance of *B. fragilis* in tumor samples (Wang et al., 2012; Zeller et al., 2014; Nakatsu et al., 2015). *B. fragilis* has been identified as an important human intestinal symbiont and has been suggested to act as a “keystone pathogen” in the development of CRC (Hajishengallis et al., 2012). *B. fragilis* is an obligate anaerobe and is a minority member of the normal colonic microbiota with a propensity for mucosal adherence (Sears et al., 2014). Previous reports have linked enterotoxigenic *B. fragilis* (ETBF) to human diarrheal illnesses and increased tumorigenesis in an IL-23-dependent and STAT3-dependent manner (Wick et al., 2014). The toxin fragylisin, produced by ETBF, is a zinc-dependent metalloprotease that triggers NF-kB signaling and cleaves E-cadherin, and has been suggested to be oncogenic (Wu et al., 2009). Bacterial genera known for their role in butyrate production, such as *Ruminococcus, Roseburia*, and *Butyricimonas* were more abundant among rectal-cancers, differing from results reported so far. An explanation for this difference could involve the fact that most data has been derived from fecal samples and/or grouping different anatomical tumor sites (such as proximal, distal, and rectal). An OTU assigned to *Bilophila*, a bile-resistant, strictly anaerobic bacterial genus, was also more abundant among rectal-cancer samples, and evidence suggests that products of bacterial bile acid conjugation, secondary bile acids, are carcinogenic (McGarr et al., 2005; Ridlon et al., 2014). *Desulfovibrio*, a commensal sulfate-reducing bacterium, may contribute to mucosal inflammation through hydrogen sulfide production, a resulting by-product of sulfated mucin metabolism (Earley et al., 2015). *Phascolarctobacterium*, known to produce propionate via succinate fermentation, was also increased among cancer samples. On the other hand, we found that *L. delbrueckii* was more abundant in non-cancer samples. Probiotic *Lactobacilli* can modify the enteric flora and are thought to have a beneficial effect on enterocolitis. Treatment of IL-10-deficient mice with the probiotic *Lactobacillus salivarius* ssp. reduced the intensity of mucosal inflammation and the incidence of colon cancer from 50 to 10%. These effects were accompanied by significant reductions in fecal coliform, enterococci, and *Clostridium perfringens* levels (O'Mahony et al., 2001). This study exemplifies the effect of changes at the flora level on the development of inflammation, and supports the hypothesis that there are “protective” species and “harmful” species in the normal bacterial flora.

After identifying relevant cancer-related microorganisms, the next steps of microbiome studies will certainly involve microbial manipulations to reduce disease-associated agents, or increase the frequency of protective and health-associated microbes. This can be achieved through diet, exemplified by a previous study using animal models that showed taurine consumption lead to a reduction of *Proteobacteria* (especially *Helicobacter*), as well as an elevation in short-chain fatty acids (SCFA) and a reduction in fecal lipopolysaccharides (LPS) (Yu et al., 2016). Duque et al., recently demonstrated, using SHIME® (Simulator of the Human Microbial Ecosystem), that the consumption of non-pasteurized fresh orange juice was able to significantly increase levels of *Lactobacillus* spp., *Enterococcus* spp., *Bifidobacterium* spp., and *Clostridium* spp. and to reduce enterobacteria (Duque et al., 2016).

Long before associations between cancer and the microbial flora started to be uncovered, diet recommendations—including low consumption of red meat and fat, and high ingestion of fibers and vegetables—have been recognized as protective against the development of colorectal cancer. Current evidences suggest that diet recommendations may be effective, together with tissue environment and host-related factors, because they also help shape the gut microbiota (Sonnenburg and Bäckhed, 2016). Further research may show that treatment of rectal dysbiosis may contribute to the prevention of inflammation-induced rectal carcinoma development and aid in chemotherapy and overall treatment response (Yang and Pei, 2006).

## Author contributions

Conceived and designed the experiments: AT, EJ, AL, SA, DN, ED; Performed the experiments: AT, EJ, RR, PC; Analyzed the data: AT, CH, DN, JS, ED; Contributed reagents/samples/analysis tools: MB, AL, SA, AC, HF, IS; Wrote or edited the manuscript: AT, HF, CH, JS, DN, ED. All authors read and approved the final manuscript.

## Funding

AT was supported by a fellowship from FAPESP (2015/01507-7). CH was supported by CAPES grant (88887.062078/2014-00) and FAPESP grant (2013/07914-8). This project was supported by PRONON (SIPAR 25000.055.167/2015-23), by Associação Beneficiente Alzira Denise Hertzog Silva (ABADHS) and by CAPES grant 3385/2013.

### Conflict of interest statement

The authors declare that the research was conducted in the absence of any commercial or financial relationships that could be construed as a potential conflict of interest. The reviewer DAS and handling Editor declared their shared affiliation and the handling Editor states that the process nevertheless met the standards of a fair and objective review.

## Supplementary material

### Availability of supporting data

Nucleotide sequences used for this study have been deposited in the SRA under accession SRP077097.
