# Supplementary material for: Tissue-Associated Bacterial Alterations in Rectal Carcinoma Patients Revealed by 16S rRNA Community Profiling
Source: Front Cell Infect Microbiol. 2016 Dec 9;6:179. doi: 10.3389/fcimb.2016.00179 (PMC5145865; doi:10.3389/fcimb.2016.00179)
Supplement: Supplementary file 1 [file DataSheet1.docx]

**Supplementary Tables**

**Supplementary Table 1** – ***Coverage analysis of the V4-V5 16S rRNA primers.*** Percentage of entries for each bacterial phyla capable of being amplified by the primer pair used in this study using two distinct databases.

|  | **SILVA Database** | **RDP database** |
| --- | --- | --- |
| **Taxonomy** | **Coverage (%)** | **Coverage (%)** |
| *Acidobacteria* | *91.9* | *32.2* |
| *Actinobacteria* | *87.4* | *64.0* |
| *Firmicutes* | *86.5* | *62.2* |
| *Proteobacteria* | *86.1* | *48.6* |
| *Eubacteria* | *84.4* | *52.1* |
| *Bacteroidetes* | *84.1* | *46.8* |
| *Spirochaetes* | *76.1* | *59.3* |
| *Lentisphaerae* | *46.3* | *62.2* |
| *Verrucomicrobia* | *21.6* | *10.9* |

The releases of the SILVA (115) and the RDP (11.2) databases used for this analysis contained, respectively, 621,948 and 2,518,232 16S rRNA sequences.

**Supplementary Table 2 – ANOSIM and ADONIS P-values for beta diversity metrics**

| **Variable** | **Bray-Curtis** | **Weighted UniFrac** | **Unweighted UniFrac** |
| --- | --- | --- | --- |
| Cancer Status | **0.001** | **0.001** | **0.001** |
| Age | 0.174 | 0.084 | 0.416 |
| Gender | 0.39 | 0.467 | 0.765 |
| Alcohol Use | 0.837 | 0.616 | 0.821 |
| Tobacco Use | 0.443 | 0.317 | 0.511 |
| BMI | 0.328 | 0.346 | 0.215 |
| Library Construction | 0.079 | 0.162 | 0.053 |

**Supplementary Table 3 – Significantly altered eubacterial genera**

| **NC average log abundance** | **RC average log abundance** | **P-value** | **Adjusted p-value** | **Genera** |
| --- | --- | --- | --- | --- |
| 2.72786 | 3.93961 | <0.00001 | 0.00002 | *Bacteroides* |
| 0.83565 | 0 | <0.00001 | 000005 | *Gordonia* |
| 1.25775 | 0.05973 | <0.00001 | 0.00007 | *Mycobacterium* |
| 1.87651 | 0.04899 | <0.00001 | 0.00007 | Streptophyta;Other |
| 0.82514 | 0.02198 | <0.00001 | 0.00008 | Bradyrhizobiaceae;Other |
| 1.98092 | 0.62536 | <0.00001 | 0.00009 | *Propionibacterium* |
| 0.87774 | 0.05345 | <0.00001 | 0.00013 | *Gammaproteobacteria*;Other;Other |
| 2.6611 | 1.76745 | <0.00001 | 0.00015 | *Flavobacteriaceae*;Other |
| 2.47629 | 1.77602 | 0.00001 | 0.00019 | *Pedobacter* |
| 2.97225 | 2.13766 | 0.00001 | 0.00019 | Caulobacteraceae;Other |
| 3.92932 | 3.13342 | 0.00001 | 0.00019 | Caulobacteraceae;Other |
| 1.16188 | 0.0884 | 0.00001 | 0.00028 | *Corynebacterium* |
| 0.65707 | 0.04495 | 0.00002 | 0.00033 | *Methylobacteriaceae*;Other |
| 0.02436 | 0.71369 | 0.00002 | 0.00036 | *Bacteria;OD1;ZB2* |
| 2.7997 | 1.97207 | 0.00002 | 0.00037 | *Stenotrophomonas* |
| 0.78585 | 0.07745 | 0.00003 | 0.00052 | *Cupriavidus* |
| 1.54013 | 2.68062 | 0.00003 | 0.00053 | *Oscillospira* |
| 0.52366 | 0.03423 | 0.00004 | 0.00055 | *Micrococcus* |
| 0.37147 | 0 | 0.00005 | 0.00069 | *Sphingobium* |
| 1.1198 | 0.48911 | 0.00005 | 0.00071 | *Cryocola* |
| 0.67971 | 1.78009 | 0.00012 | 0.00127 | *Parabacteroides* |
| 0.97814 | 0.26582 | 0.00012 | 0.00132 | *Delftia* |
| 0.63127 | 1.82232 | 0.00011 | 0.00135 | *Phascolarctobacterium* |
| 0.60592 | 0 | 0.00013 | 0.00139 | *Pseudoxanthomonas* |
| 1.72703 | 1.04623 | 0.00011 | 0.0014 | *Phyllobacterium* |
| 0.19251 | 1.03978 | 0.00015 | 0.00149 | *Sphingobacteriales*;Other;Other |
| 1.87862 | 1.06379 | 0.00016 | 0.0015 | *Achromobacter* |
| 1.02134 | 0.2965 | 0.00022 | 0.00205 | *Agrobacterium* |
| 4.36791 | 3.64157 | 0.00024 | 0.00219 | *Pseudomonas* |
| 0.34173 | 0.96061 | 0.00027 | 0.00234 | *Butyricicoccus* |
| 2.02432 | 1.457 | 0.00028 | 0.00238 | *Ralstonia* |
| 0.0118 | 0.4539 | 0.00034 | 0.00277 | *Bilophila* |
| 1.06488 | 0.41083 | 0.00045 | 0.00344 | *Bosea* |
| 1.8036 | 1.18288 | 0.00044 | 0.00345 | *Microbacterium* |
| 2.20322 | 1.12309 | 0.00052 | 0.00383 | *Lactobacillus* |
| 1.45505 | 0.65716 | 0.00052 | 0.00383 | *Leptothrix* |
| 2.22609 | 3.25283 | 0.00066 | 0.00465 | *Proteobacteria*;Other |
| 0.42341 | 0 | 0.00081 | 0.00541 | *Alphaproteobacteria;BD7-3*;Other |
| 0.517 | 1.43419 | 0.00081 | 0.00553 | Mogibacteriaceae;Other |
| 1.69821 | 2.56372 | 0.00099 | 0.00641 | Lachnospiraceae;Other |
| 0.53578 | 1.50052 | 0.00111 | 0.00702 | *Odoribacter* |
| 0.46891 | 0.09128 | 0.00123 | 0.00758 | *Staphylococcus* |
| 0.04355 | 0.54074 | 0.00137 | 0.00831 | *Anaerotruncus* |
| 1.74882 | 2.62796 | 0.00164 | 0.00967 | *Ruminococcus* |
| 0 | 0.35395 | 0.00188 | 0.01053 | *Eggerthella* |
| 0.37566 | 0 | 0.00188 | 0.01053 | *Bacteroidetes;VC2_1_Bac22*;Other;Other |
| 2.5775 | 3.45617 | 0.00185 | 0.01068 | *Rickettsiales;mitochondria*;Other |
| 1.10861 | 2.13944 | 0.00203 | 0.01101 | *Ruminococcaceae*;Other |
| 0.92306 | 0.4004 | 0.00251 | 0.01332 | *Paracoccus* |
| 0.41266 | 0.02425 | 0.00266 | 0.01381 | *Bacillus* |
| 0.03798 | 0.41391 | 0.00345 | 0.01723 | *Dehalobacterium* |
| 0.50455 | 0.13249 | 0.00341 | 0.01737 | Xanthomonadaceae;Other |
| 0.0679 | 0.62329 | 0.00441 | 0.01881 | *Butyricimonas* |
| 0.81836 | 0.34735 | 0.0045 | 0.01889 | *Devosia* |
| 2.09557 | 1.35514 | 0.00464 | 0.01917 | *Rhodococcus* |
| 0.2843 | 0.03304 | 0.00392 | 0.01921 | *Sphingomonas* |
| 0.18049 | 0 | 0.00421 | 0.01922 | *Brevibacterium* |
| 0 | 0.36006 | 0.00421 | 0.01922 | *Holdemania* |
| 0.4206 | 0 | 0.00421 | 0.01922 | *Phycisphaerales*;Other |
| 0.25781 | 0 | 0.00421 | 0.01922 | *Phenylobacterium* |
| 0.30905 | 0 | 0.00421 | 0.01922 | *Alcaligenes* |
| 0 | 0.17648 | 0.00421 | 0.01922 | *Salinispora* |
| 0.21616 | 0 | 0.00421 | 0.01922 | Neisseriaceae;Other |
| 0.64114 | 0.15547 | 0.00498 | 0.02024 | *Methylobacterium* |
| 0.51148 | 0.27749 | 0.00551 | 0.02206 | *Klebsiella* |
| 1.20959 | 0.6059 | 0.00581 | 0.0229 | *Ochrobactrum* |
| 0.01969 | 0.37693 | 0.00643 | 0.02496 | Pseudoramibacter_Eubacterium |
| 1.00661 | 1.89353 | 0.00749 | 0.02823 | *Clostridium* |
| 1.02624 | 1.96762 | 0.00743 | 0.02841 | *Ruminococcaceae;* |
| 0.30401 | 0.94955 | 0.00794 | 0.02907 | *Anaerostipes* |
| 0.54555 | 0.06668 | 0.00789 | 0.02929 | *Neisseria* |
| 0.7725 | 0.30852 | 0.00819 | 0.02959 | *Chitinophaga* |
| 0.43109 | 0.14865 | 0.00835 | 0.02972 | *Mesorhizobium* |
| 0.93075 | 1.86605 | 0.00889 | 0.02984 | *Rikenellaceae* |
| 0.6996 | 1.3803 | 0.00889 | 0.02984 | *Alphaproteobacteria*;Other |
| 1.12924 | 0.5645 | 0.00852 | 0.02995 | *Acinetobacter* |
| 0.13628 | 0.71245 | 0.00877 | 0.03001 | *Desulfovibrio* |
| 0 | 0.29329 | 0.00913 | 0.03004 | *Rikenellaceae;Other* |
| 2.37599 | 1.47944 | 0.00871 | 0.03021 | *Escherichia* |
| 0.36068 | 0.03171 | 0.00932 | 0.03029 | *Brevundimonas* |
| 1.39391 | 2.31612 | 0.00963 | 0.03091 | *Roseburia* |
| 0.77581 | 0.32226 | 0.01233 | 0.0391 | *Anaerococcus* |
| 1.30108 | 2.1394 | 0.01418 | 0.04443 | *Lachnospira* |
| 1.11987 | 0.53975 | 0.01561 | 0.04721 | *Chitinophagaceae*;Other |
| 0.58827 | 0.19767 | 0.01561 | 0.04774 | *Rubrobacter* |
| 1.46959 | 2.15151 | 0.01557 | 0.0482 | *Clostridiales*;Other;Other |

**Supplementary Table 4 – Genera/OTUs associated with lymph node status**

| **Taxonomic Level** | **P-value** | **R^2^** | **Positive samples in both groups** | **Taxonomy** |
| --- | --- | --- | --- | --- |
| Genera | 0.00409 | 0.41191 | 4 | Firmicutes; Clostridia; Clostridiales; Lachnospiraceae; Oribacterium |
| Genera | 0.04528 | 0.22762 | 8 | Firmicutes; Clostridia; Clostridiales; Mogibacteriaceae; Mogibacterium |
| OTU | 0.01377 | 0.32349 | 5 | Proteobacteria; Alphaproteobacteria; Rickettsiales; mitochondria |
| OTU | 0.02241 | 0.28531 | 5 | Firmicutes; Clostridia; Clostridiales; Ruminococcaceae; Ruminococcus; flavefaciens |
| OTU | 0.02209 | 0.28645 | 7 | Bacteroidetes; Saprospirae; Saprospirales; Chitinophagaceae; |
| OTU | 0.02163 | 0.28813 | 3 | Tenericutes; RF3; ML615J-28 |
| OTU | 0.02286 | 0.28372 | 15 | Firmicutes; Clostridia; Clostridiales; Lachnospiraceae; Dorea |
| OTU | 0.02247 | 0.28509 | 6 | Proteobacteria |
| OTU | 0.00811 | 0.36326 | 6 | Firmicutes; Clostridia; Clostridiales |
| OTU | 0.01294 | 0.32829 | 12 | Firmicutes; Clostridia; Clostridiales; Ruminococcaceae |
| OTU | 0.04182 | 0.23428 | 14 | Firmicutes; Clostridia; Clostridiales; Lachnospiraceae; Roseburia; faecis |
| OTU | 0.04314 | 0.23167 | 4 | Proteobacteria; Alphaproteobacteria; Rickettsiales; mitochondria |
| OTU | 0.02971 | 0.26250 | 11 | Firmicutes; Clostridia; Clostridiales; Peptostreptococcaceae; |
| OTU | 0.02247 | 0.28510 | 5 | Firmicutes; Clostridia; Clostridiales ;Lachnospiraceae; Dorea |
| OTU | 0.03805 | 0.24214 | 5 | Firmicutes; Clostridia; Clostridiales; Mogibacteriaceae; Mogibacterium |
| OTU | 0.02781 | 0.26790 | 3 | Proteobacteria |
| OTU | 0.04766 | 0.22332 | 6 | Firmicutes; Clostridia; Clostridiales; Lachnospiraceae |
| OTU | 0.04438 | 0.22930 | 10 | Proteobacteria; Alphaproteobacteria; Caulobacterales; Caulobacteraceae |
| OTU | 0.04009 | 0.23780 | 9 | Proteobacteria; Alphaproteobacteria; Rickettsiales; mitochondria |
| OTU | 0.03990 | 0.23819 | 9 | Firmicutes; Clostridia; Clostridiales; Lachnospiraceae; Roseburia |
| OTU | 0.03153 | 0.25765 | 9 | Firmicutes; Clostridia; Clostridiales; Ruminococcaceae |
| OTU | 0.03694 | 0.24459 | 17 | Firmicutes; Clostridia; Clostridiales; Lachnospiraceae; Coprococcus; catus |
| OTU | 0.04155 | 0.23480 | 5 | Firmicutes; Clostridia; Clostridiales |
| OTU | 0.01681 | 0.30803 | 5 | Proteobacteria; Alphaproteobacteria; Rickettsiales; mitochondria |
| OTU | 0.00409 | 0.41191 | 4 | Firmicutes; Clostridia; Clostridiales; Lachnospiraceae; Oribacterium |
| OTU | 0.04583 | 0.22661 | 4 | Firmicutes; Clostridia; Clostridiales; Ruminococcaceae; Ruminococcus |

**Supplementary Figures**

**Supplementary Figure 1.** Cluster structure recovered from relative abundances of genera level counts using four distance metrics. Cluster quality was tested using: (A) prediction strength, (B) silhouette index and (C) Caliński-Harabasz statistic.

**Supplementary Figure 2.** Boxplots showing log abundances for the 12 most abundant phyla. Phyla with significant differences are labeled with (*). Significant p-values were found for *Cyanobacteria*, *Actinobacteria*, *Bacteroidetes*, *OD1* (P-values < 0.001), *Proteobacteria* (P-value = 0.002) and *Planctomycetes* (P-value = 0.002).

**Supplementary Figure 3.** High-level phenotypical differences between microbe proportions. The phenotypical differences include proportion of aerobic/anerobic, gram-positive/gram-negative and biofilm forming bacteria. P-values were calculated by the Wilcoxon rank sum test. Middle vertical lines represent mean proportional abundance and outer vertical lines represent interquartile ranges.

**Supplementary Figure 4.** Boxplots showing log abundances for the four top OTUs significantly associated with lymph node positive rectal-cancer samples.
