# Supplementary figures and images for: Tissue-Associated Bacterial Alterations in Rectal Carcinoma Patients Revealed by 16S rRNA Community Profiling
Source: Front Cell Infect Microbiol. 2016 Dec 9;6:179. doi: 10.3389/fcimb.2016.00179 (PMC5145865; doi:10.3389/fcimb.2016.00179)

**A**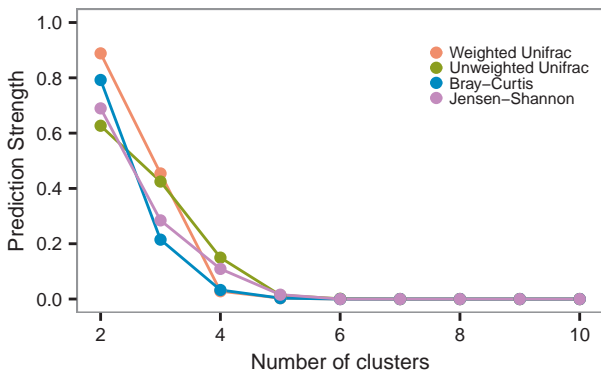**B**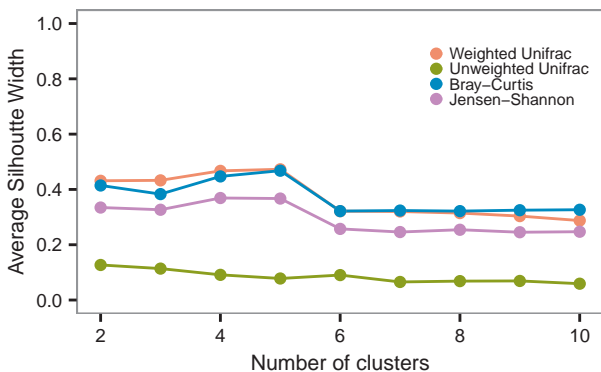**C**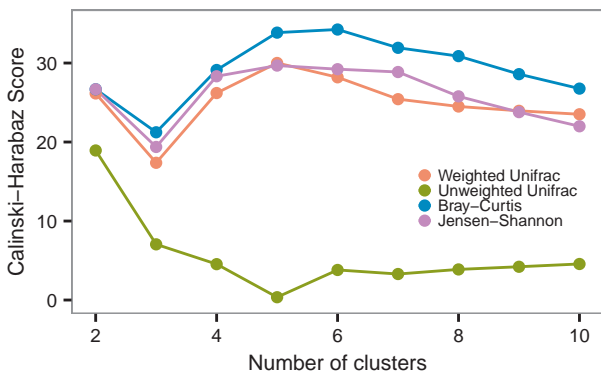

Supplement: Supplementary file 2 [file Image1.PDF]

Log Abundance

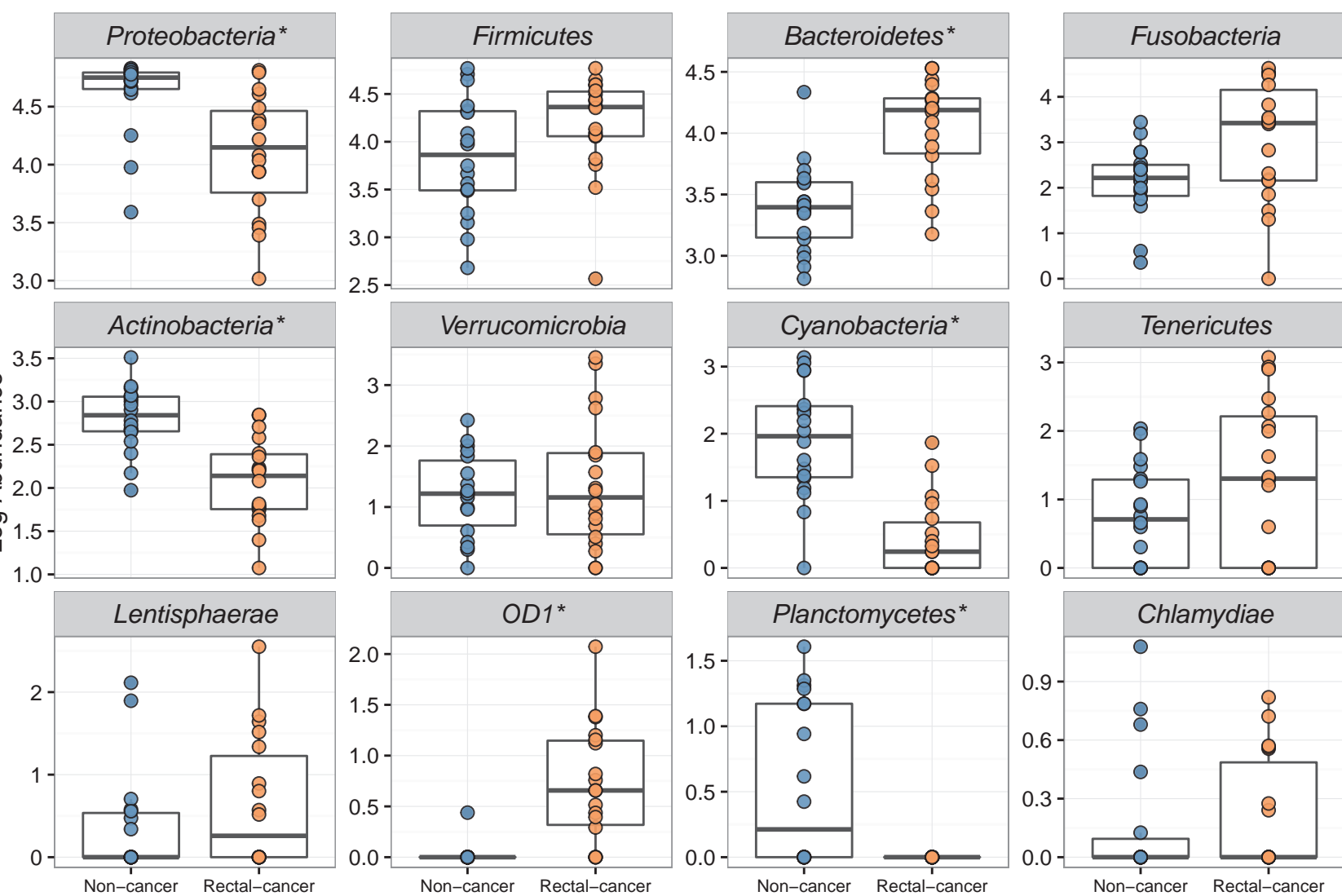

Supplement: Supplementary file 3 [file Image2.PDF]

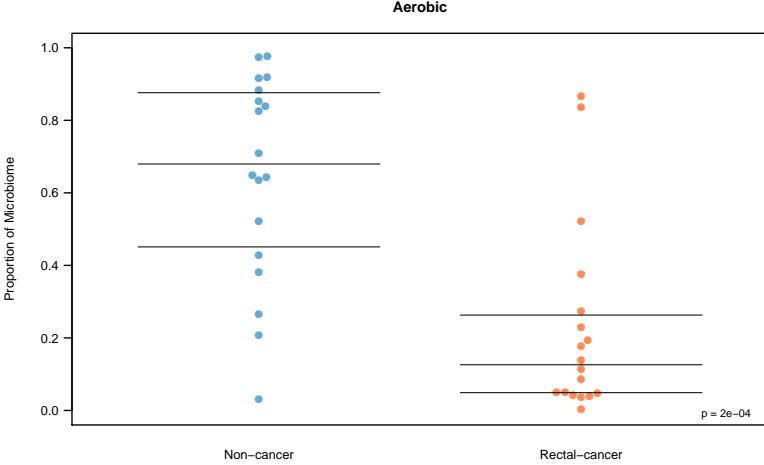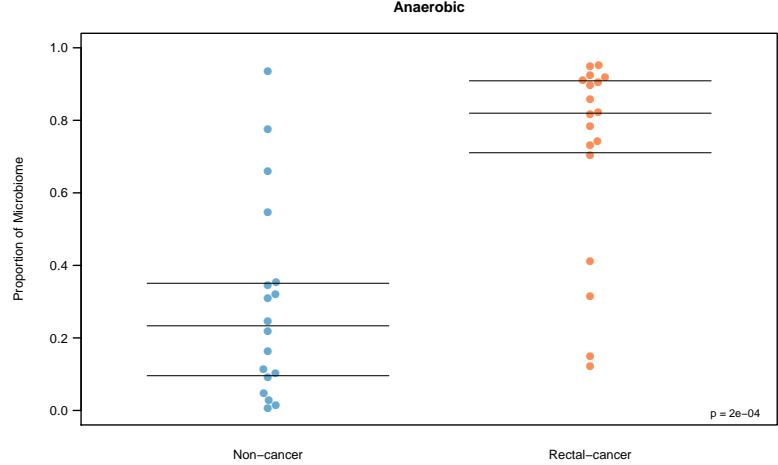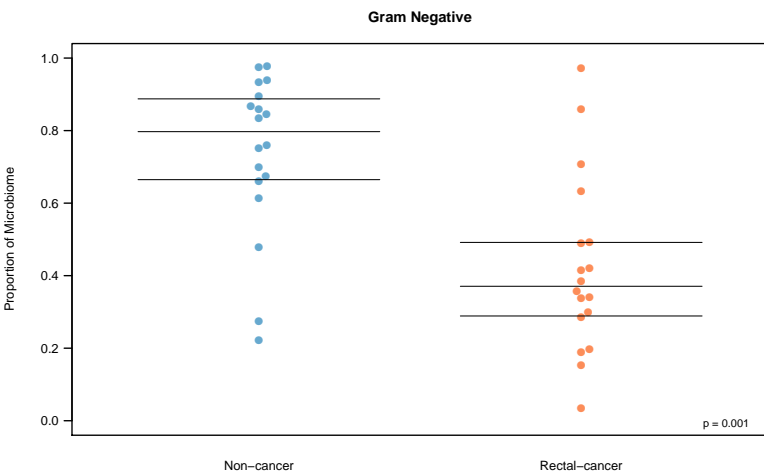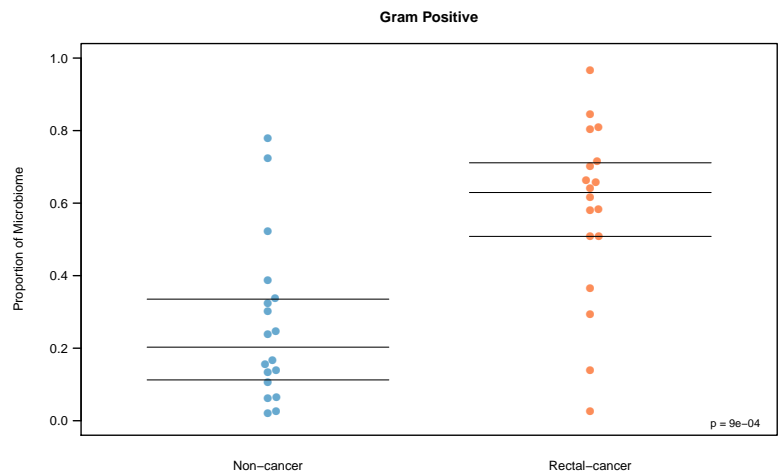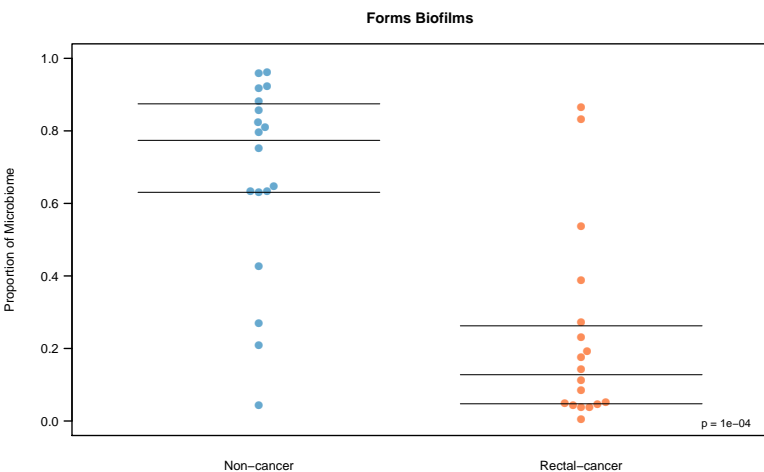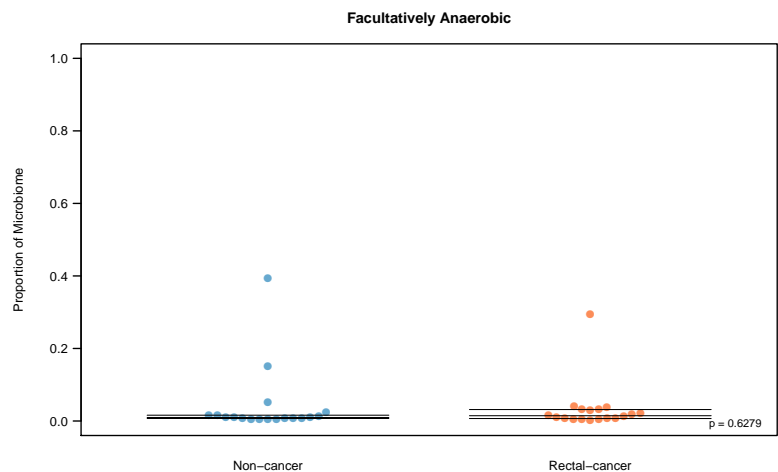

Supplement: Supplementary file 4 [file Image3.PDF]

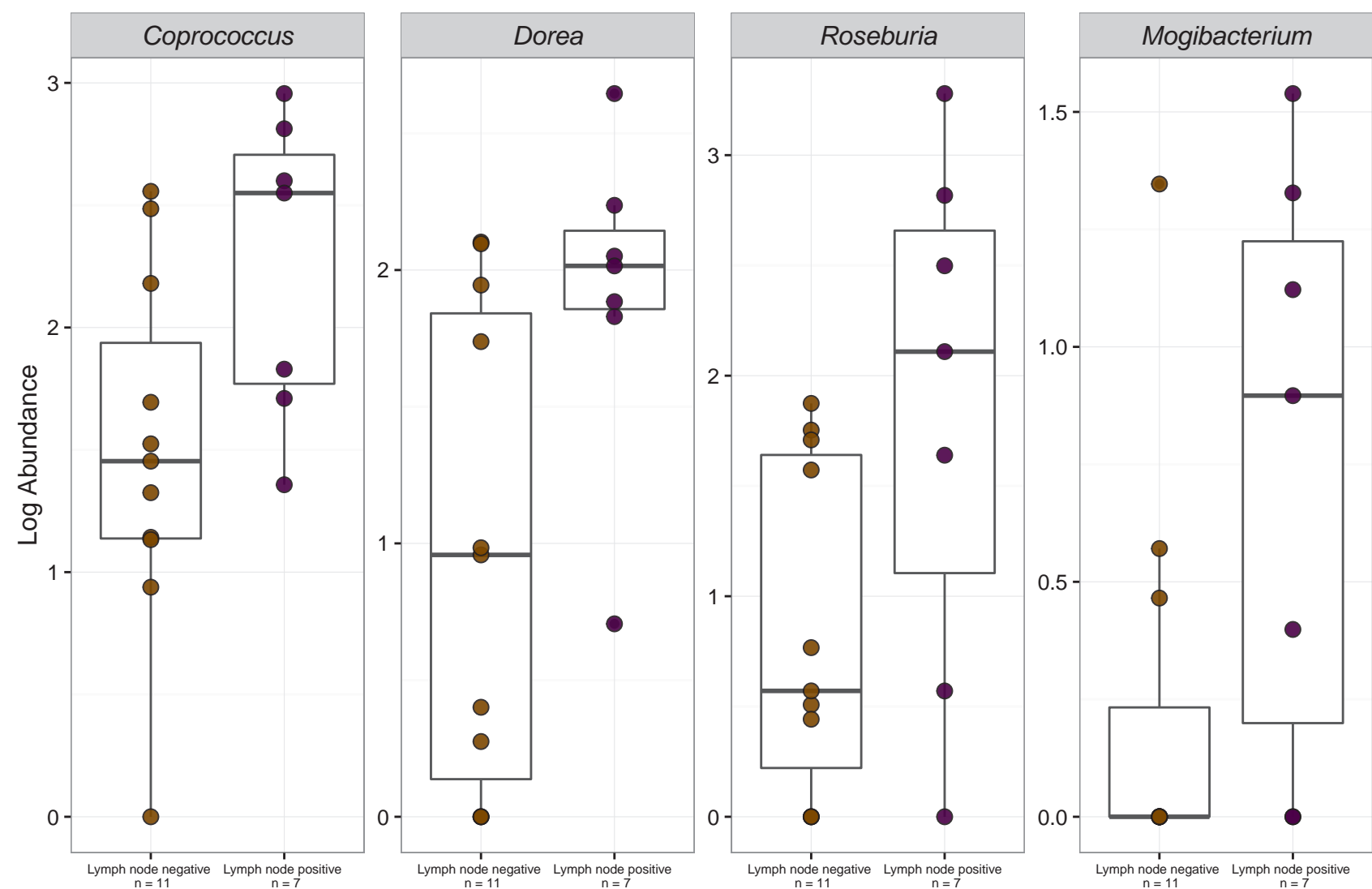

Supplement: Supplementary file 5 [file Image4.PDF]
